# Editorial: Integrated approaches to understanding and improving poultry health, immunity, and productivity: unraveling the role of metabolism

**DOI:** 10.3389/fphys.2026.1844387

**Published:** 2026-04-21

**Authors:** Felix Kwame Amevor, Victoria Anthony Uyanga, Sandra G. Velleman, Colin G. Scanes

**Affiliations:** 1 Department of Animal Science, Shandong Agricultural University, Taian, Shandong, China; 2 Department of Agriculture and Environmental Sciences, Lincoln University of Missouri, Jefferson City, MO, United States; 3 Department of Animal Sciences, The Ohio State University, Wooster, OH, United States; 4 Department of Biological Science, University of Wisconsin-Milwaukee, Milwaukee, WI, United States

**Keywords:** gut-liver axis, immune functioning, kupffer cells, liver, microbiome

The needs of modern poultry production extend beyond the traditional objectives of maximizing growth rate and feed efficiency, requiring an understanding of the complex and dynamic interplay among biological systems that collectively determine productivity and resilience in poultry. Central to this is nutrient metabolism, which governs the transformation of dietary substrates into the biochemical intermediates required for cellular processes, physiological regulation, and systemic homeostasis. The Research Topic “*Integrated Approaches to Understanding and Improving Poultry Health, Immunity, and Productivity: Unraveling the Role of Metabolism*” brings together novel contributions that aim to enhance nutrient utilization, modulate gut microbiota, improve metabolic resilience, and support immune competence.

The gut-liver axis consists of the gastrointestinal tract (GIT), GIT microbiota, and liver, encompassing hepatocytes and Kupffer cells (reviewed: Albillos et al., 2020; Tilg et al., 2022; Pabst et al., 2023). These are linked via the hepatic portal blood vessels with “*bidirectional crosstalk*” (reviewed: Tilg et al., 2022). Implicit to this axis are the intestinal barrier and Kupffer cells, the resident macrophages in the liver sinusoids (reviewed: Albillos et al., 2020; Tilg et al., 2022; Pabst et al., 2023). Factors affecting the axis include the GIT microbiome, microbial metabolites, nutrients, other feed supplements, and pathogens (reviewed: Albillos et al., 2020; Tilg et al., 2022; Pabst et al., 2023).

In a landmark review, Amevor et al. concluded that “*the liver-gut axis represents a cornerstone of poultry health and productivity*” with the gut microbiome and its metabolites being pivotal to liver metabolism and immunity, while the bile produced by the liver helps to shape the gut microbiota. The functioning of the liver-gut axis can be improved by:

- The use of probiotics, prebiotics, and synbiotics to enhance microbial diversity and liver function. For instance, both *Lactobacillus* and Bifidobacterium species can enhance the microbial generation of short-chain fatty acids (SCFAs), which help to reduce hepatic inflammation.
- Optimal inclusion of specific amino acids and micronutrients in the feed, such as methionine, which is required for hepatic detoxification and glutathione synthesis, and zinc to improve gut membrane integrity.
- Feed additives to optimize immune functioning and reduce inflammation, including omega-3 fatty acids, fiber (e.g., beta-glucans), and phytogenic additives such as oregano oil and curcumin.
- Genomics to identify molecular markers and genetic selection for gut barrier integrity.
- *In ovo* nutritional interventions may also contribute to the early programming of gut-liver interactions and immune development.

Oke et al. discussed the concept of immunometabolism, *i.e.*, how nutrients and microbiome-derived metabolites influence both immune and metabolic function. These microbial metabolites, including SCFAs such as acetate, propionate, and butyrate, play an important role in maintaining intestinal epithelial integrity, modulating inflammatory pathways, and providing energy substrates for enterocytes. In addition, the microbial transformation of bile acids produces secondary bile acids, such as deoxycholic acid and lithocholic acid, which influence host lipid metabolism and metabolic signaling pathways. This evidence points to the fact that gut microbiota-derived metabolites are directly involved in regulating both the immune and metabolic systems of poultry.

Usman et al. examined the effects of fermented tropical forages, specifically two cultivars of elephant grass (*Cenchrus purpureus* cv. Guiminyin and cv. Purple), on the cecal microbiota of broiler chickens. Their findings demonstrate the potential of fermented forages as an alternative feed resource capable of partially replacing corn or soybeans in chicken feed with fermented cultivars of the elephant grass. The inclusion of these fermented cultivars promoted the enrichment of microbial taxa associated with fiber degradation and SCFA production, thereby supporting a diverse and functionally resilient gut microbial ecosystem.

Li et al. concluded that heat stress and lipopolysaccharide challenges act to “*dysregulate whole-body metabolism*”. There were increases in body temperature, oxygen consumption, carbon dioxide expiration, and heat production in broiler chickens subjected to heat stress. In contrast, a lipopolysaccharide challenge reduced the metabolic rate and heat production in chickens. The authors also demonstrated that dietary L-citrulline may play a protective role in maintaining physiological homeostasis under stress conditions.

Sulaiman et al. examined the effects of probiotics, prebiotics, and synbiotics on growth performance and immune organ development in turkeys. Between 10 and 16 weeks of age, dietary supplementation with probiotics or synbiotics significantly enhanced growth performance. In addition, dietary treatment with either probiotics or synbiotics increased the relative weight of immune organs, including the bursa of Fabricius and the spleen, which suggests enhanced immune competence alongside improved productivity.

Bittencourt et al. reported on the linkage between circulating concentrations of total carotenoids and body weight gain in poultry. Their work suggests that plasma carotenoid levels may serve as useful biomarkers for evaluating productive performance and assessing the efficacy of feed additives, particularly supplements that are thought to improve intestinal barrier integrity and nutrient absorption.

Dos Anjos et al. examined the effects of either heat treatment or enzymatic supplementation of cowpeas (*Vigna unguiculata*) or pigeon peas (*Cajanus cajan*) as a soybean replacement in broiler diets. The performance of birds receiving feeds containing cowpeas or pigeon peas was inferior to that of birds receiving soybeans. However, when chicks received feeds containing cowpeas to replace soybeans, there were increments in apparent dry matter digestibility and apparent nitrogen digestibility if the cowpeas had been subject to heat treatment or there was enzymatic supplementation of the feeds. There was a decrease in the viscosity of ileal contents in chickens fed roasted rather than raw pigeon peas or cowpeas.

Gobbo et al. conducted a long-term exploratory study to evaluate how preventative health programs implemented at various stages of broiler breeder production would support the health status of commercial broiler breeders. Their findings indicate that nutraceutical-based health management strategies could prevent disease occurrence, enhance resilience to disease challenges, and maintain the production performance of broiler parent flocks.

Importantly, the gastrointestinal tract is modulated by the enteric nervous system, gut hormones, and neuropeptides. Scanes et al. examined the release of ghrelin and the expression of ghrelin genes in the crop, proventriculus, and duodenal explants from young chicks *in vitro*. There were marked effects of cholinergic antagonists on both the release of ghrelin and the expression of the ghrelin genes, ghrelin O-acyltransferase genes, and growth hormone secretagogue receptor genes in the crop, proventriculus, and duodenum. This is consistent with cross-talk between the enteric nervous system and gastrointestinal endocrine tissues.

In summary, this Research Topic presents innovative approaches on how poultry health, immunity, and productivity can be improved by taking a closer look at the underlying role of metabolism. The contributions to this Research Topic use multidisciplinary frameworks to address poultry nutrition, physiology, immunology, genomics, welfare, and the adoption of integrated methodologies encompassing the monitoring of productive performance, nutrient-gene interactions, digestibility assessments, metabolic profiling, biochemical assays, gut histopathological assessments, and microbiota analysis to gain in-depth knowledge and a holistic understanding of poultry metabolism and productivity.
